# The Potential of Laser-Light Backscattering for Assessment of Physicochemical and Oxidative Stress-Related Traits in Arouquesa Beef

**DOI:** 10.3390/ani16172665

**Published:** 2026-08-25

**Authors:** Mariana Caipira Lei, Mariana Almeida, Virgínia Santos, José António Silva, José Manuel Almeida, Luís Félix, Severiano Silva, Carlos Venâncio

**Affiliations:** 1 Animal and Veterinary Department, University Institute of Health Sciences (IUCS), Polytechnic and University Higher Education Cooperative (CESPU), Avenida Central de Gandra, 1317, 4585-116 Gandra, Portugal; 2 Centre for the Research and Technology of Agro-Environmental and Biological Sciences (CITAB), Inov4Agro, University of Trás-os-Montes and Alto Douro (UTAD), Quinta de Prados, 5000-801 Vila Real, Portugal; lfelix@utad.pt (L.F.); cvenanci@utad.pt (C.V.); 3 Veterinary and Animal Research Centre (CECAV) and Associate Laboratory of Animal and Veterinary Science (AL4AnimalS), University of Trás-os-Montes e Alto Douro, Quinta de Prados, 5000-801 Vila Real, Portugal; mdantas@utad.pt (M.A.); vsantos@utad.pt (V.S.); jasilva@utad.pt (J.A.S.); malmeida@utad.pt (J.M.A.); ssilva@utad.pt (S.S.); 4 Department of Animal Science, School of Agrarian and Veterinary Sciences (ECAV), University of Trás-os-Montes and Alto Douro (UTAD), Quinta de Prados, 5000-801 Vila Real, Portugal; 5 Department of Veterinary Science, University of Trás-os-Montes and Alto Douro (UTAD), Quinta de Prados, 5001-801 Vila Real, Portugal

**Keywords:** backscattering images, beef colour, DFD beef, meat quality monitoring, oxidative stress biomarkers, pH

## Abstract

The beef industry is interested in quick, non-destructive methods to assess meat quality. This study evaluated the potential of laser-light backscattering imaging (LLBI) for assessing physicochemical properties and oxidative stress-related traits in Arouquesa beef, exploring its applicability as a non-invasive approach for detecting alterations potentially associated with pH. In 34 samples of *Longissimus thoracis et lumborum* (*LTL*) muscle from Arouquesa steers, the pH and oxidative stress biomarkers were quantified 24 h post-mortem, and backscatter images were obtained using green and red laser sources, with the areas corresponding to the core, inner ring, and outer ring of the diffusion patterns measured. After 7 days’ storage of the samples under vacuum at 4 °C, the measurements of oxidative stress biomarkers were repeated, and colour, water-holding capacity, sarcomere length (SL), and shear force (SF) were quantified. Significant correlations were found between LLBI parameters and pH 24 h post-mortem (pH_24h_), colour coordinates, cooking losses (CL), drip losses (DL), and SF. Moreover, oxidative stress biomarkers also correlated with laser scattering parameters. Overall, LLBI demonstrated considerable potential as a rapid and non-invasive technique for assessing physicochemical quality attributes and oxidative stress-related traits in Arouquesa beef, supporting its application as an innovative tool for meat quality monitoring.

## Simple Summary

Dark, firm, and dry beef is a major meat quality defect that reduces product value and consumer acceptance. It results from pre-slaughter stress, which alters muscle metabolism and oxidative stress, and ultimately affects the physicochemical properties of meat. Although laser-light backscattering imaging has shown potential for non-destructive meat assessment, its ability to detect optical changes associated with physicochemical and oxidative alterations related to dark, firm, and dry beef has not been investigated. Therefore, this study evaluated the relationships among laser-light backscattering imaging measurements, post-mortem pH, meat quality traits, and oxidative stress indicators in the *Longissimus thoracis et lumborum* muscle of Arouquesa cattle. Laser-light backscattering imaging parameters showed significant associations with pH, colour, cooking losses, drip losses, shear force, antioxidant activity, and oxidative status. These findings demonstrate the potential of laser-light backscattering imaging as a rapid, non-destructive tool for identifying stress-related changes associated with dark, firm, and dry beef and support its further development for meat quality assessment.

## 1. Introduction

Meat quality is a critical attribute in the beef industry, influencing consumer acceptance, shelf life, and economic value [1].

Beef colour and tenderness are the most important traits in consumers’ choice and purchase and are determinants for palatability and for a positive eating experience [2,3]. Dark meat is one of the main characteristics that consumers find unappealing, resulting in financial losses for the meat production sector [1].

With slaughter, the abrupt cut of oxygen supply to tissues and the inhibition of mitochondrial respiration lead to oxidative stress that can alter beef quality traits [4]. Oxidative stress significantly influences the quality, shelf life, nutritional value, and appearance of beef. It occurs when the production of free radicals exceeds the animal’s antioxidant defence capacity [5] and damages muscle proteins, lipids, and pigments that are known to significantly affect both meat texture and visual appearance by altering the tissue’s structural and optical properties [6,7]. The levels of reactive oxygen species (ROS), and therefore oxidative stress, are higher when the animals are exposed to higher stress levels before slaughter [4]. In beef cattle, this can happen during growth, transport, heat stress, disease, poor nutrition, or after slaughter [5].

Indeed, pre-slaughter stress has a significant impact on beef quality by altering muscle metabolism, accelerating glycogen depletion, and affecting the decline in post-mortem pH [1]. Pre-slaughter stress depletes muscle glycogen reserves, which limits the post-mortem production of lactic acid [8,9]. These phenomena are responsible for the major meat quality defect associated with pre-slaughter stress: Dark, firm, and dry (DFD) meat. This condition is characterised by a high ultimate pH, dark colour, firm texture, and reduced water loss [1]. As a result, DFD meat presents challenges for both processors and consumers due to its unattractive appearance and reduced microbiological stability [1,10]. Oxidative stress post-mortem may also aggravate these results [6,7]. Thus, DFD meat prediction may be used to identify compromised welfare, as it is directly related to animal stress [4].

The pH measurement is the conventional method for identifying meat affected by DFD. However, it is a semi-destructive procedure, which represents a methodological limitation [11] and is not the only trustworthy marker of DFD meat. Oxidative stress determination in meat is also limited by the complexity of laboratory work, including expensive equipment, time-consuming analyses, and specialised technical expertise [12]. Therefore, there is currently no accurate, non-destructive method for the rapid assessment of biomarkers associated with meat quality [13].

In recent years, non-destructive optical techniques have gained attention as promising alternatives for rapid meat quality evaluation, particularly those that offer accessible solutions given the economic limitations of local industries in implementing advanced technologies [14]. Among these, LLBI has emerged as a valuable tool due to its sensitivity to changes in tissue structure, as well as optical properties related to water content, muscle fibre arrangement, biochemical composition, pH, colour, tenderness, and microbial spoilage [15,16,17,18,19]. LLBI uses a laser beam that strikes the surface of a sample and then is scattered back and captured by a detector that reads the intensity pattern of the scattered light. The backscattering profile is a product of the interaction of light photons with the sample constituents and surface pattern and consequent scattering. Therefore, changes in sample structure and composition can modify the intensity and spatial distribution of the backscattered light [20]. This technique has been studied across several agricultural and food products, including for the assessment of drying, ripening, firmness, colour, maturity, defects from handling and chilling, and compositional or structural changes, demonstrating its potential for rapid and non-destructive quality assessment [21]. However, the potential of LLBI to detect alterations in meat associated with oxidative stress-related traits remains largely unexplored.

The Arouquesa cattle breed is a typical indigenous breed reared under a traditional mountain farming system in northern and central Portugal [22]. As is common with other breeds in similar circumstances, this breed requires the quality of its meat to be promoted to continue making its socio-economic contribution to the region in which it is found. Recently, as part of efforts to characterise its meat and improve the production system, it has been found that it has a slightly elevated pH [23].

Consequently, this study aimed to evaluate the potential of the laser-light backscattering technique for identifying physicochemical and oxidative stress-related meat traits in Arouquesa beef, thereby contributing to the development of innovative, non-invasive tools for meat quality control in traditional and high-value beef production systems.

## 2. Materials and Methods

### 2.1. Animals and LTL Muscle Samples

Thirty-four Arouquesa male steers (298 ± 38 days) were slaughtered at a certified abattoir. After slaughter, carcasses (149 ± 25 kg) were dressed, split into two halves, and chilled for 24 h at 2 °C. In addition, from one half-carcass of each animal, a lumbar cut from the first to the fifth vertebrae was obtained. All cuts were refrigerated and transferred from the slaughterhouse to the laboratory for subsequent analyses. From each cut, one sample of *LTL* was obtained, totalling thirty-four different muscle samples. More details on the Arouquesa breed production system can be found in the work of Sacarrão-Birrento et al. [22].

The *LTL* muscle samples were collected 24 h after slaughter, then vacuum-packaged and aged for 7 days at 4 °C. pH measurements were taken at 24 h, and backscatter images using green and red laser sources were also obtained at this time to assess their predictive potential in relation to meat quality. Biomarkers of oxidative stress were assessed at both 24 h and 7 days to understand their progression, whilst the physicochemical parameters of colour, water-holding capacity, SL and SF were assessed after 7 days of storage, as this is when the meat becomes available to the consumer.

### 2.2. Meat Quality Traits

#### 2.2.1. pH

The pH was measured using a penetration electrode connected to a pH meter (WTW 330i, Weilheim, Germany) previously calibrated with buffers at pH 4.01 and 7.00. Measurements taken within 24 h post-mortem are commonly used as an indicator of meat quality, with a pH value of 5.8 or higher generally accepted as a threshold [24,25]. Following the criteria established by Silva et al. [24], the meat samples were classified as Normal (pH < 5.8, *n* = 15) or indicative of DFD meat (pH > 5.8, *n* = 19).

#### 2.2.2. Colour

The surface colour measurements of the meat were obtained using a Minolta Chroma Meter CR-310 colorimeter (Konica Minolta, Osaka, Japan), which assesses colour in the L*, a*, and b* three-dimensional colour space, as defined by CIE Lab [26]. In this system, L* represents luminosity, while a* indicates the red-green component, and b* represents the yellow-blue component. The colour was measured on the surface of the meat after a 60-min blooming period. During these 60 min, the samples were placed in trays covered with polyethylene film and kept at 4 °C [27]. Prior to use, the colorimeter was calibrated with a standard white ceramic plate under D65 illumination.

#### 2.2.3. Drip Losses

DL were calculated by subtracting the final weight (Wf) from the initial weight (Wi) at day seven (D7) post-mortem, following seven days of vacuum-packaged storage at 4 °C [28]. These losses were expressed as a percentage of the Wi.

$$DL\;(\%)=\frac{Wi-Wf}{Wi}\times 100$$

#### 2.2.4. Cooking Losses

Samples weighing approximately 90–100 g were measured and placed in a polyethylene bag. These bags were then submerged in a Digiterm 100 water bath (JP Selecta, Barcelona, Spain) set to 80 °C until the internal temperature of the samples reached 71 °C, as monitored by a thermocouple. After reaching the desired temperature, each sample was transferred to ice water until its internal temperature dropped to 15 °C. The samples were then stored for about 24 h at 4 °C. Once removed from the bags, the meat was carefully dried and weighed to determine the Wf. CL were calculated as the difference between the Wi and the Wf after cooking, expressed as a percentage of the Wi [28]:

$$CL\;(\%)=\frac{Wi-Wf}{Wi}\times 100$$

These samples were subsequently packaged and stored at 4 °C to be used in SF determination.

#### 2.2.5. Sarcomere Length

SL was evaluated using the method described in Cross et al. [29]. Briefly, approximately 2 g of *LTL* was dissected into small pieces using a scalpel and suspended in 30 mL of cold 0.25 M sucrose solution, and subsequently homogenised at a slow speed for 60 s with the Ultra-Turrax T25B (Kika Labortechnik, Staufen, Germany). A drop of the homogenate was placed on a slide using a Pasteur pipette, covered with a coverslip, and observed under the Nikon Labophot-2 (Nikon, Tokyo, Japan) optical microscope in phase 1, using the 40× objective with an attached camera. The length of 10 consecutive sarcomeres was measured, and approximately 15 groups of 10 sarcomeres per sample were measured using the image analysis software Matrox Inspector 4.1 (Matrox Electronic Systems Ltd., Dorval, QC, Canada). The average length of the sample sarcomeres was subsequently determined.

#### 2.2.6. Shear Force

Meat samples used to determine the CL were removed from the refrigerator and were cut into cuboid-shaped sub-samples (6 to 8) of 1 cm^2^ cross-section and 3–4 cm in length with muscle fibres parallel to the length of the cuboid. After reaching room-temperature equilibrium, the sub-samples were placed with fibres perpendicular to the direction of a Warner–Bratzler rectangular hole probe coupled to a TA.XT.plus texturometer (Stable Micro Systems, Godalming, UK), with a load cell of 30 kg.f., blade velocity set to 200 mm/min, and trigger force of 5 g. Maximum SF was recorded, and the values were expressed in N/cm^2^.

### 2.3. Oxidative Stress-Related Traits

The representative muscle samples of *LTL* were weighed and homogenised for 30 s with 0.5 mL of ice-cold HEPES buffer adjusted to pH 7.4 and supplemented with sucrose (0.32 mM), MgCl_2_ (1 mM), and PMSF (0.5 mM) using a VWR VDI 12 homogeniser (Radnor, PA, USA). The samples were then centrifuged at 12,000× *g* for 10 min at 4 °C, and the supernatants were collected, and protein was determined at 280 nm.

Superoxide dismutase (SOD) activity was determined according to Durak et al. [30] at 560 nm. Samples were incubated in potassium phosphate buffer containing hypoxanthine, EDTA, and NBT, followed by the addition of xanthine oxidase. Activity was quantified using SOD standards (0–60 U/mL) and expressed as U/mg protein. Catalase (CAT) activity was measured according to Claiborne [31] at 240 nm by monitoring H_2_O_2_ decomposition in sodium phosphate buffer. Results were quantified using CAT standards (0–6 U/mL) and expressed as U/mg protein. Glutathione peroxidase (GPx) activity was determined according to Massarsky et al. [32] at 340 nm by monitoring NADPH oxidation (ε = 6.22 mM^−1^cm^−1^). Samples were incubated with sodium phosphate buffer containing EDTA, NaN_3_, NADPH, glutathione reductase, and GSH, followed by the addition of H_2_O_2_. Results were expressed as µmol NADPH/min/mg protein. GST activity was determined according to Habig and Jakoby [33] at 340 nm using CDNB as substrate (ε = 9.60 mM^−1^cm^−1^). Samples were incubated with potassium phosphate buffer containing CDNB and GSH. Results were expressed as µmol CDNB/min/mg protein. Reduced glutathione (GSH) activity levels were determined according to Gartaganis et al. [34] using OPT fluorescence (Ex 320 nm/Em 420 nm). Samples were deproteinised with 40% TCA and centrifuged, and the supernatant was reacted with OPT in phosphate buffer containing EDTA. Quantification was performed using GSH standards (0–1000 µM), and results were expressed as µmol GSH/mg protein. Oxidised glutathione (GSSG) activity levels were determined according to Gartaganis et al. [34] using OPT fluorescence (Ex 320 nm/Em 420 nm). Samples were incubated with NEM to derivatise GSH, followed by the addition of NaOH and OPT. Quantification was performed using GSSG standards (0–1000 µM), and results were expressed as µmol GSSG/mg protein. The OSI was calculated as the ratio between GSH and GSSG. Total oxidant status (TOS) was determined according to Erel [35] at 560 nm. Samples were incubated with xylenol orange reagent, followed by the addition of ferrous ammonium sulphate/o-dianisidine solution. Absorbance was measured after 5 min and quantified using an H_2_O_2_ standard curve (0–1 mM). ROS levels were determined according to Deng et al. [36] using DCFH-DA fluorescence (Ex 485 nm/Em 530 nm). Briefly, 20 µL of the sample was mixed with PBS and DCFH-DA (10 mg/mL in DMSO), incubated for 30 min at 37 °C, and fluorescence was measured. Quantification was performed using a DCF standard curve (0–100 µM), and the results were expressed as µmol DCF/mg protein. Thiobarbituric acid reactive substances (TBARS) were determined according to Wallin et al. [37] at 530 nm (MDA-TBA adducts) and 600 nm (non-specific absorbance). Samples were incubated with phosphate buffer, BHT, TBA, and TCA for 40 min at 60 °C and cooled on ice. Quantification was performed using MDA standards, and results were expressed as µmol MDA/mg protein.

### 2.4. LLBI System

**Figure 1:**
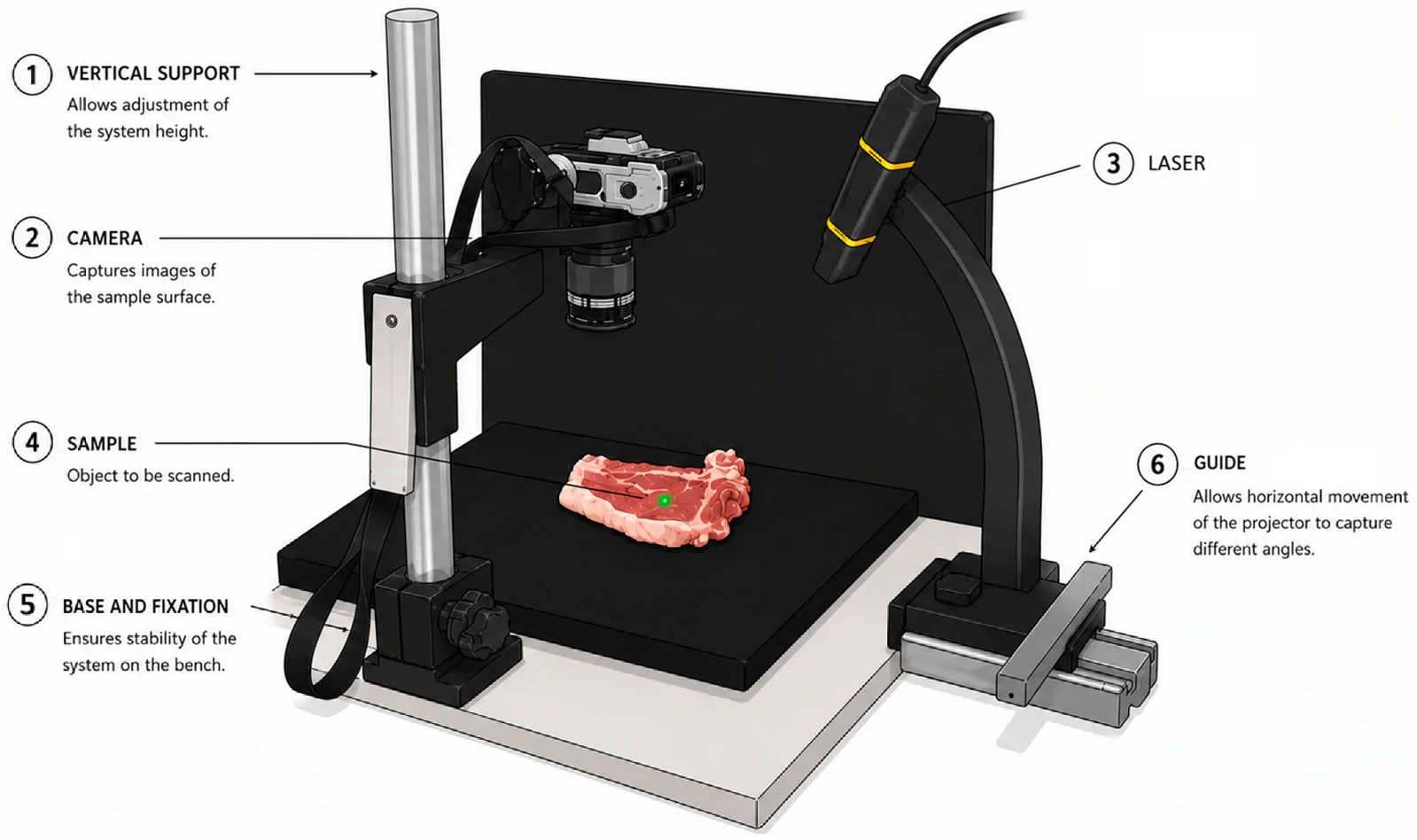
Illustrative diagram of the custom-built imaging system and technique used to obtain the LLBI.

LLBI was acquired using a custom-built imaging system developed at the University of Trás-os-Montes and Alto Douro (UTAD) (Figure 1). The system was specifically designed for laser light backscattering imaging applications in biological tissues and was optimised through preliminary testing on different meat samples during its development. These preliminary evaluations, although not published, were used to establish the imaging geometry, illumination conditions, and image acquisition protocol adopted in the present study, ensuring stable and repeatable image acquisition. The system comprised two main components: (1) two laser diodes, including a green laser (530 nm, 5 mW, 1 mm beam diameter) and a red laser (635 nm, 5 mW, 1 mm beam diameter), divergence < 1 mrad, which were applied separately to the sample surface; (2) an Olympus EM-5 (Tokyo, Japan) digital camera equipped with a 60 mm OM macro lens and a circular polarising filter, positioned at a distance of 30 cm from the *LTL* samples, capturing images with a resolution of 4640 × 3472 pixels. The laser beam was directed onto the sample surface at an incidence angle of 75°, with cross-sectioned *LTL* samples placed on a non-reflective black background under standardised illumination conditions. Image acquisition was performed inside a dark chamber to minimise interference from ambient light. Images were collected from sample areas free of visible defects or surface irregularities. The backscattered light patterns generated by the interaction between the laser beam and the sample were recorded by the camera and subsequently transferred to a computer for image processing and quantitative analysis.

#### Backscattering Image Analysis

**Figure 2:**
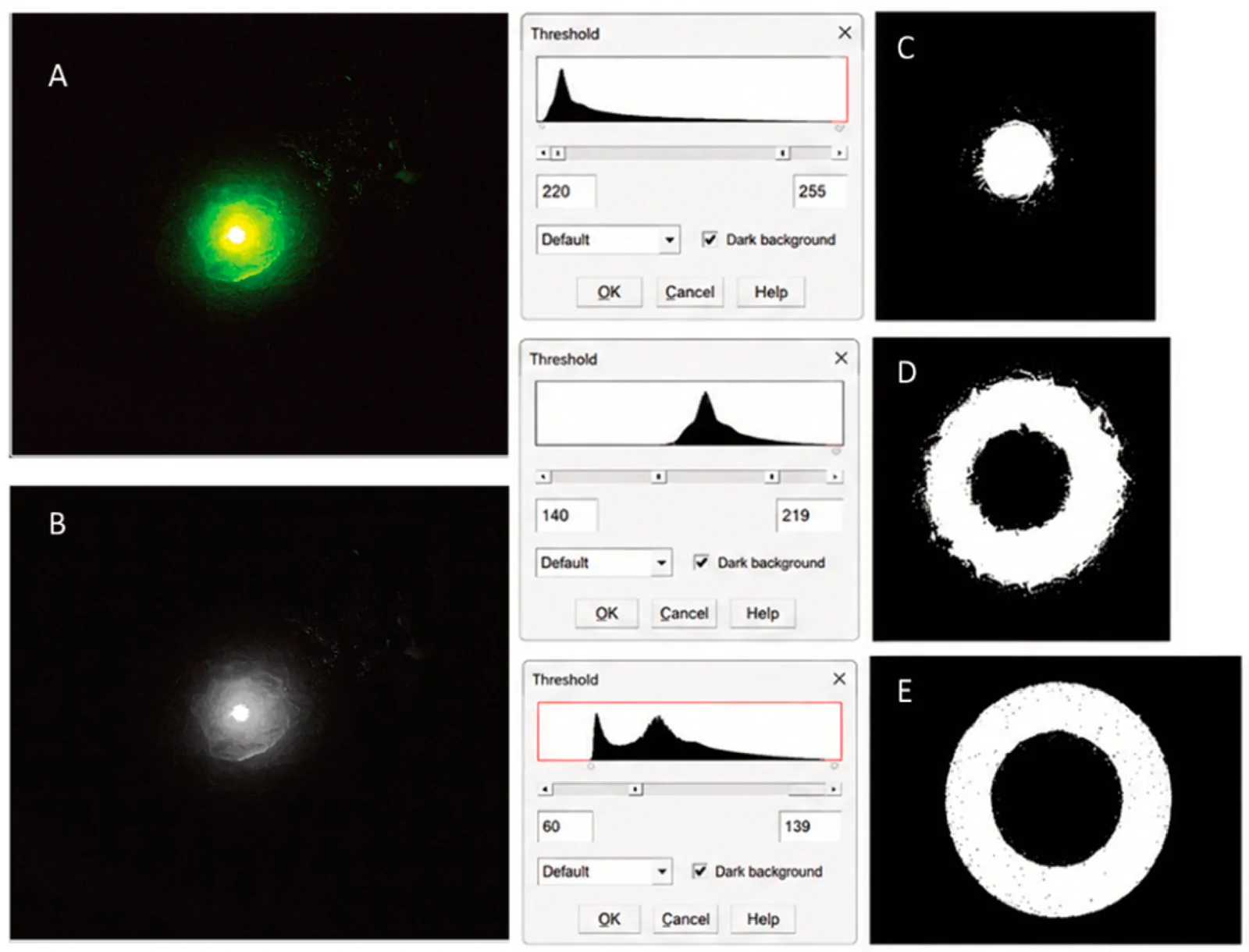
Workflow used for image processing and segmentation in Fiji/ImageJ. The original image (**A**) was converted to an 8-bit grayscale format prior to segmentation (**B**). Thresholding was applied to isolate three concentric central regions according to pixel intensity ranges: (**C**) bright white core (220–255), (**D**) bright inner ring (140–219), and (**E**) outer ring (60–139). Masks were generated for each segmented region, and the corresponding area was quantified.

The LLBI exhibited radial symmetry characterised by three concentric circular regions, with a highly intense central bright area. Image processing and quantitative analysis were performed using Fiji (ImageJ 1.49u). For both green and red laser images, three regions of interest (ROIs) were defined: the core region, the inner ring, and the outer ring (Figure 2). Accordingly, in the green laser images, the green core area (GCA), green inner area (GIA), and green outer area (GOA) were measured. Likewise, in the red laser images, the red core area (RCA), red inner area (RIA), and red outer area (ROA) were determined.

### 2.5. Statistical Analysis

Unless otherwise specified, all measurements were performed in triplicate for each sample, and the average was used for subsequent statistical analyses. All data were screened for outliers by the ROUT method. No outliers corresponding to extreme biologically implausible values or suggestive of methodological errors were identified. Therefore, no observations were excluded from the analysis. All collected data on physicochemical and stress-related meat traits, as well as the laser-light backscattering area measurements, were subjected to a descriptive statistical analysis. The normality of each variable was assessed using the Shapiro–Wilk test. Changes in oxidative stress biomarkers between Day 1 (D1) and D7 were analysed using paired Student’s *t*-tests. As all variables were normally distributed and the relationships between variables were approximately linear, Pearson’s correlation coefficient (r) was used to assess the strength and direction of the associations between physicochemical and stress-related meat traits and the laser-light backscattering area measurements. Statistical significance was declared at *p* < 0.05. To account for multiple testing, *p*-values from the correlation analyses were adjusted using the Benjamini–Hochberg procedure to control the false discovery rate (FDR), with statistical significance established at an FDR-adjusted *p* < 0.05. All the data were analysed using JMP software (JMP, Pro 17, SAS Institute, Hong Kong, China).

### 2.6. Animal Welfare Disclaimer

All conditions and procedures performed with the animals were recognised as common animal husbandry practices that took place according to Portuguese (Decreto-Lei 113/2013) and EU (Directive 2010/63/EU) legislation for animal experimentation and welfare. The procedures were carried out upon approval by the UTAD animal experimentation committee under reference 1507-e-DZ-2020.

## 3. Results and Discussion

### 3.1. Characteristics of Backscattering Images

**Figure 3:**
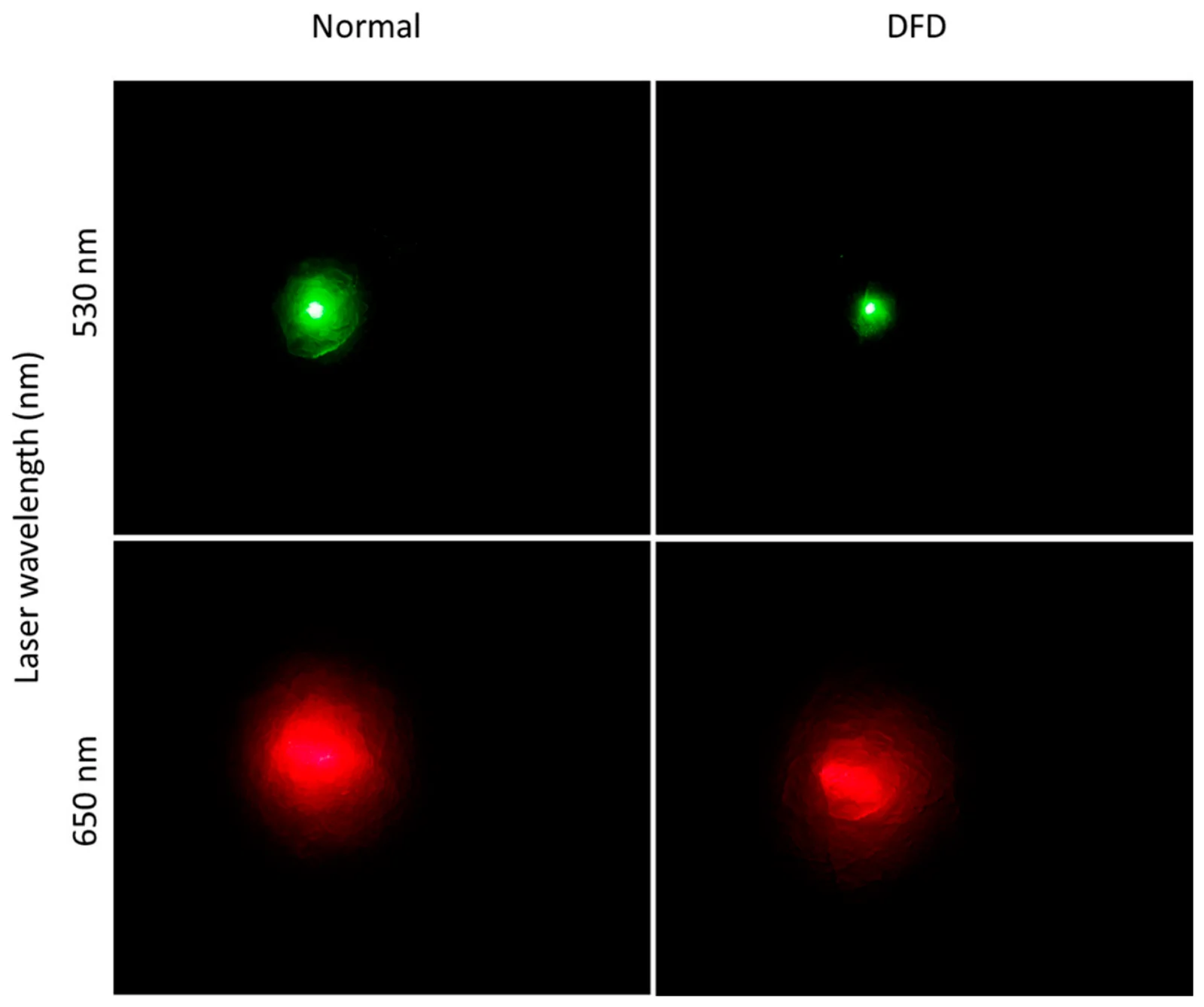
Comparison of backscattering images between a normal *LTL* sample and a dark, firm, and dry (DFD) *LTL* sample at different wavelengths. The areas in green show the light-scattering produced by the green laser, and those in red show the light-scattering produced by the red laser, for both normal meat and DFD meat.

In backscattering imaging, the characteristics of meat affect the light scattering pattern, thereby determining the reflectance detected by the imaging system. Figure 3 shows the differences in the intensity distributions of backscattering patterns between a normal and a DFD *LTL* sample. The red-light laser produced a larger light-scattering area than the green-light laser, likely due to its longer wavelength and deeper penetration into muscle tissue, which enhances photon diffusion and scattering within the meat structure [38]. The absorption of light by the meat samples was higher for the green laser (532 nm) than for the red laser (635 nm) because biological tissues, including meat, contain pigments such as myoglobin and, to a lesser extent, haemoglobin that absorb shorter wavelengths (green light) more strongly than longer wavelengths (red light), resulting in greater attenuation of the green laser [39]. Visually, normal meat consistently showed a wider perimeter than DFD across both wavelengths. This result was observed in other studies, which reported a decrease in scattering area as water content decreased [17,40]. These results show that laser wavelength influences light scattering and detection sensitivity in backscattering imaging.

### 3.2. Descriptive Statistics

Table 1 summarises the descriptive statistics for physicochemical meat quality traits, stress-related biomarkers, and laser-light backscattering area measurements obtained from Arouquesa *LTL* samples. Mean values, standard deviation (SD), minimum and maximum values, and coefficients of variation (CV) were calculated to characterise the variability and distribution of the evaluated parameters.

Descriptive statistics for physicochemical meat traits, stress-related biomarkers, and laser-light backscattering area measurements demonstrated substantial variability among the evaluated parameters, reflecting differences in meat quality characteristics, oxidative status, and optical responses. Regarding physicochemical traits, pH_24h_ showed a mean value of 6.18 with relatively low variability (CV = 7.90). However, the observed range (5.49–6.98) indicates substantial variation among samples despite the relatively low CV. Meat colour parameters presented moderate CVs, with L* and a* showing CV values of 11.4%, while b* exhibited greater variability (CV = 34.20%). Water-holding capacity traits displayed high dispersion, particularly DL (CV = 54.30%) and CL (CV = 40.40%), suggesting marked differences in muscle water retention among samples. SF also showed high variability (CV = 44.50%), indicating considerable variation in meat tenderness, whereas SL remained relatively stable (CV = 8.00%).

For oxidative stress-related biomarkers measured at D1, antioxidant and oxidative stress indicators showed moderate to high variability. OSI and GPx exhibited the highest CV (80.54% and 70.50%, respectively), suggesting substantial differences in oxidative metabolism among animals. Conversely, GSH (CV = 15.00%) and ROS (CV = 17.70%) presented lower variability at D7, indicating greater consistency in redox balance parameters on that day.

Laser-light backscattering area measurements also exhibited considerable variability. Among the green laser parameters, the GIA showed the highest CV (67.80%), whereas the GCA demonstrated lower variability (47.50%). Similarly, red laser measurements presented high dispersion, particularly for the RCA and ROA, with CV values of 58.00% and 54.40%, respectively. These findings indicate substantial heterogeneity in light scattering behaviour among samples, likely associated with differences in muscle structure, water distribution, and physicochemical composition.

Overall, the descriptive analysis demonstrated that both meat quality traits and laser backscattering measurements exhibited sufficient variability to support correlation analyses, highlighting the potential of laser-light backscattering as a tool for characterising physicochemical and stress-related attributes in Arouquesa beef.

### 3.3. Correlations Between Laser-Light Backscattering Area and Physicochemical Traits

Table 2 presents the correlation coefficients between laser-light backscattering area parameters and physicochemical meat quality traits measured in Arouquesa beef. The analysis was performed to evaluate the relationships between optical scattering measurements obtained with green and red lasers and physicochemical attributes associated with meat quality.

Correlation analysis revealed significant relationships between laser backscattering parameters and physicochemical meat quality traits, demonstrating the optical technique’s ability to reflect variations in muscle quality characteristics. Stronger correlations were generally observed for pH_24h_, colour parameters, DL, CL, and SF, whereas SL showed weak or negligible associations with laser measurements.

Among the evaluated traits, ultimate pH_24h_ exhibited the strongest negative correlations with both green and red laser parameters. The red laser measurements showed particularly high coefficients, ranging from −0.89 to −0.91, indicating a highly significant inverse relationship between pH_24h_ and laser scattering areas. Similar negative correlations were observed for the green laser measurements (−0.62 to −0.76). These results suggest that lower pH_24h_ values were associated with increased light scattering, likely due to protein denaturation, reduced water-holding capacity, and alterations in water distribution and muscle microstructure within the tissue, which are known to affect the optical properties of meat [41,42].

On the other hand, meat colour parameters (L*, a*, and b*) demonstrated positive correlations with laser area measurements. The strongest associations were observed for the RIA, particularly for yellowness (b*; r = 0.73) and redness (a*; r = 0.71), indicating that brighter, more intensely coloured meat samples had larger scattering areas. The larger scattering areas observed in brighter and more intensely coloured meat samples are consistent with previous findings showing that increased muscle light scattering and reflectance contribute to enhanced meat lightness and colour intensity through changes in muscle structure, optical properties, and microstructural modifications of muscle fibres [42,43]. Green laser measurements also showed moderate to strong positive correlations with colour traits, especially for lightness (L*; r = 0.73). These findings indicate that laser-light backscattering is sensitive to the optical and structural properties associated with meat colour development, since muscle microstructure, myofilament lattice spacing, and light-scattering behaviour are known to influence meat lightness and colour perception [44].

Water-holding capacity traits, represented by DL and CL, also showed significant positive correlations with laser parameters. CL presented some of the highest coefficients among all physicochemical traits, with values reaching 0.76 for the GIA and 0.76 for the RIA. DL was more strongly associated with red laser measurements, particularly ROA (r = 0.65). The observed laser scattering patterns appear to reflect changes in muscle water distribution and structural organisation. These patterns were likely influenced by both water mobility and the integrity of the muscle microstructure, as alterations in water distribution and myofibrillar organisation are known to affect light-scattering properties in meat tissues [42,45].

SF showed moderate positive correlations with both laser wavelengths, with the strongest association observed for RIA (r = 0.57). This indicates that tougher meat samples tended to exhibit greater light-scattering areas, potentially reflecting differences in muscle fibre organisation and structural integrity, since muscle microstructure and myofibrillar arrangement are known to influence both meat tenderness and light-scattering behaviour [41,42]. In contrast, SL presented very weak correlations with all laser measurements (−0.06 to 0.17), suggesting limited sensitivity of the laser backscattering technique to variations in this structural parameter. However, these weak correlations for SL may be influenced by low variability in the studied samples (CV = 8.00%). Overall, these findings demonstrate that laser-light backscattering may provide a reliable, non-destructive approach for assessing physicochemical meat quality traits in Arouquesa beef.

Previous studies have demonstrated that laser-light scattering measurements are associated with beef quality traits such as pH, colour, and tenderness and may serve as useful inputs for predictive models [46,47]. Therefore, the backscattering parameters found could potentially be combined through calibration models to predict laboratory-measured meat quality traits [48]. More complex machine-learning approaches could also be explored with larger datasets.

### 3.4. Correlations Between Laser-Light Backscattering Area and Oxidative Stress-Related Traits

There is growing interest in understanding the oxidative process in meat and the parameters that characterise it, with a view to their potential use as biomarkers of meat quality [49,50,51,52]. Particular attention has been paid to antioxidant capacity parameters and their effects on lipid oxidation and colour [51,53,54], yet the relationship with light backscattering has been poorly explored [44,52].

In beef with a high pH, a higher mitochondrial content and an abundance of Krebs cycle metabolites have been reported, which indicate greater mitochondrial oxygen consumption. Consequently, the reduced availability of oxygen for muscle maturation leads to the development of dark-coloured muscle [51,55]. Moreover, at high pH, dark muscle shows greater Z-disc degradation, higher sarcoplasmic protein activity, and increased water-holding capacity of myofibrillar proteins, thereby inducing muscle fibre swelling and shrinkage of the space between fibrils, which may contribute to lower light scattering [56]. In contrast, the normal oxidative process in meat develops more readily when muscle pH is low, there are more spaces between the cells and muscle bundles in the structure, and moisture content is higher. Consequently, light backscattering measurements are also higher [44].

Table 3 presents the correlation coefficients between laser-light backscattering area parameters and oxidative stress-related meat traits measured at D1 and D7 post-mortem in Arouquesa beef.

Overall, the results indicated that several laser-derived area parameters were moderately to strongly correlated with oxidative stress-related traits, supporting the sensitivity of laser-light backscattering to physiological and biochemical alterations associated with pre- and post-mortem stress responses.

The strongest correlations observed varied across different regions of the core, the inner ring, and the outer ring for the various oxidative stress parameters, suggesting that these image regions are sensitive to variations in muscle metabolic state and structural organisation. In general, stronger associations were observed for glutathione-related parameters, GST activity, and TOS, whereas CAT, GPx, and TBARS showed weak or negligible correlations with laser-derived measurements. Previous studies have demonstrated that the antioxidant defence system in post-mortem muscle operates as a coordinated network involving SOD, CAT, GPx, GST, and glutathione-dependent pathways [57]. CAT and GPx were the exceptions in terms of a significant association with the light parameters. Interestingly, CAT (2.74 vs. 4.38; *p* < 0.001) activity increased at D7 compared to D1. GPx activity (*p* = 0.16) showed no significant differences between storage days, as it has already been shown to remain stable during post-mortem storage, whilst significant oxidative changes were observed [53]. Moreover, these enzymes have antioxidant capacity and are dependent on H_2_O_2_ produced by SOD activity. SOD activity showed a strong positive correlation with the red laser on D1, decreasing to a weaker correlation with the same laser colour on D7, but only with the outer ring image. Concurrently, the activity of GST also reduced the strong correlation in D1, without showing any significant correlation in D7. This indicates a more unstable pattern of SOD activity [53], which may be explained by the fact that SOD (162.94 vs. 118.68; *p* < 0.001) and GST (13.74 vs. 9.16; *p* < 0.001) activity decreased over the course of the ageing period, as previously described [53], and supports the evidence of a closer relationship between GST and structural changes in the post-mortem period [58].

Still, recent evidence suggests that oxidative stress contributes to the development of DFD meat [4,52]. Differential expression of microRNAs associated with DFD beef has been linked to genes involved in glutathione metabolism and antioxidant defence, including glutathione reductase and GPX5 [59]. These findings support the importance of glutathione-dependent mechanisms in determining muscle oxidative status and provide a plausible biological framework for interpreting the correlations observed between laser backscattering measurements and glutathione-related biomarkers in the present study. The reduced form, GSH, only showed a significant correlation with GCA on D1, but this was lost on D7. The oxidised form, GSSG, increased in concentration during the ageing period (42.98 to 87.64; *p* < 0.001) and maintained the correlation observed between D1 and D7. This difference in correlation between D1 and D7 for GSH and GSSG may be due to increased GSH content during beef ageing [60] found in our study (410.24 to 555.24; *p* < 0.001), which would not be expected given the structural changes and natural oxidation that occur in the meat ageing period.

Among the parameters relating to oxidation balance in meat, the OSI shows an increased negative correlation between D1 and D7, although there is no significant variation in its values (*p* = 0.69). However, the accumulation of GSSG tends to decrease with the highest oxidative stress and the accumulation of GSSG, leading to a decreased cellular defence against free radicals during an animal’s life period or the ageing meat process [51,61]. This suggests that the brighter red colourations, associated with higher light backscattering on D1, are those that showed the greatest decrease in OSI by D7, possibly because they still had a greater oxidative reserve. The TOS reflects the balance between pro-oxidant compounds (such as ROS) and antioxidants in muscle tissue and shows a negative correlation at both D1 and D7, with identical values for both laser values. These data suggest that the lower light backscattering of dark meat corresponds to an increase in the oxidation process [52]. The increase in the oxidation process inherent to ageing explains the increase in TOS levels between D1 and D7 (1.29 to 3.44; *p* < 0.001), as previously stated [61]. The ROS levels only showed a slight correlation at D7 for both laser colours. This change may be caused by higher levels of ROS at 7 days (463.23 to 531.35; *p* < 0.01), which are associated with those samples that exhibited more intense light backscattering and a greater oxidative capacity reserve on D1 [62].

The interrelationship between lipid oxidation and myoglobin oxidation has previously been described in meat [51,55,63], with implications for meat colour and, consequently, light backscattering. The TBARS, as a current biomarker of lipid peroxidation, did not show a significant correlation with light backscattering or significant differences between D1 and D7 (*p* = 0.71). These data are in line with evidence that during the early stages of the ageing process, lipid peroxidation follows a very stable pattern, as the accumulation of lipid oxidation products is slow [64,65], a pattern that is even more pronounced in higher pH or dark meat [55,66].

These results highlight that the ageing period affected the interaction between laser light and muscle tissue, improving the ability of laser-light backscattering measurements to reflect stress-related meat quality traits.

The persistence of correlations involving GSSG and TOS at both sampling times suggests that laser backscattering may reflect broader oxidative changes occurring during post-mortem ageing. In contrast, the less consistent associations observed for ROS, SOD, and GST on D7 likely reflect the transient nature of these biomarkers and their involvement in early adaptive responses to oxidative processes. Similarly, the absence of significant correlations with TBARS may indicate that lipid peroxidation was not a major determinant of optical variation within the ageing period evaluated.

These findings highlight the potential of the LLBI technique and underscore the need for further research to assess and validate its applicability. Future studies should use larger and more diverse datasets to develop and validate predictive models, include different muscle types and animals of varying ages, and extend the ageing period to evaluate the predictive performance of LLBI at multiple post-mortem time points. The inclusion of additional parameters, such as moisture content, myoglobin oxidation, and protein carbonyls, may further contribute to a more comprehensive evaluation of the technique and its potential for meat quality assessment.

## 4. Conclusions

The results of this study suggest that LLBI measurements acquired at 24 h post-mortem are associated with pH_24h_, antioxidant activity, and several physicochemical and redox-related meat quality traits measured during ageing. These findings indicate that LLBI has potential as a rapid, non-destructive tool for assessing meat quality, although the underlying biological mechanisms linking light backscattering, oxidative processes, and post-mortem muscle changes require further clarification. This study presents promising preliminary evidence that LLBI may be a valuable tool for evaluating meat quality in Arouquesa beef. To strengthen these findings, it would be beneficial to conduct further research using larger and more diverse datasets. Incorporating predictive modelling and validating LLBI under commercial processing conditions will improve our understanding of its reliability and its ability to identify DFD-related meat quality traits. Additionally, these efforts will provide deeper insights into the role of oxidative biomarkers in the observed relationships, paving the way for more effective applications in meat quality evaluation.

## Figures and Tables

**Table 1:** Descriptive statistics of physicochemical traits, oxidative stress biomarkers, and laser-light backscattering area measurements in Arouquesa *LTL* samples.

Traits		Mean	SD	Minimum	Maximum	CV
Physicochemical meat traits						
Day 1						
pH_24h_		6.18	0.49	5.49	6.98	7.90
Day 7						
Colour	L*	37.80	4.32	31.50	49.60	11.44
	a*	23.10	2.63	19.30	27.80	11.40
	b*	6.34	2.17	3.83	10.30	34.20
DL		1.86	1.01	0.95	5.14	54.30
CL		14.70	5.94	6.77	29.60	40.40
SL		1.68	0.13	1.31	1.90	8.00
SF		56.20	25.00	19.50	130.60	44.50
Oxidative stress biomarkers						
Day 1						
SOD		162.90	42.50	98.60	267.00	26.10
CAT		2.74	0.96	0.84	4.35	34.90
GPx		5.22	3.68	0.50	14.60	70.50
GST		13.70	5.13	3.90	25.30	37.30
GSH		457.50	151.00	198.70	903.90	33.00
GSSG		42.98	14.58	20.08	73.03	33.92
OSI		6.50	5.24	2.90	30.69	80.54
TOS		1.29	0.58	0.46	2.54	44.85
ROS		463.20	121.00	271.10	805.70	26.10
TBARS		59.60	19.00	29.00	129.70	31.80
Day 7						
SOD		118.70	35.10	29.10	213.20	29.60
CAT		4.38	2.31	2.03	12.10	52.70
GPx		6.16	2.82	1.23	12.60	45.80
GST		9.17	4.99	0.41	19.30	54.40
GSH		555.20	83.20	398.80	743.80	15.00
GSSG		87.64	27.90	39.55	176.70	31.83
OSI		6.84	2.01	3.55	13.54	29.34
TOS		3.21	0.97	1.11	4.99	30.30
ROS		531.40	93.90	377.20	802.80	17.70
TBARS		64.50	17.50	22.00	101.10	27.10
Laser area measurements on day 1						
GCA		1.09	0.52	0.40	2.37	47.50
GIA		8.21	5.57	2.23	22.50	67.80
GOA		20.50	9.88	7.79	42.20	48.10
RCA		11.60	6.75	2.91	24.50	58.00
RIA		36.40	15.60	12.90	62.80	42.80
ROA		90.40	49.20	35.60	180.00	54.40

pH_24h_: pH 24 h post-mortem; DL: Drip Losses (%); CL: Cooking Losses (%); SL: Sarcomere Length (µm); SF: Shear Force (N/cm^2^); SOD: Superoxide Dismutase (U/mg protein); CAT: Catalase (U/mg protein); GPx: Glutathione Peroxidase (nmol NADH/mg protein); GST: Glutathione S-transferase (nmol CDNB/min.mg protein); GSH: Reduced glutathione (μmol GSH/mg protein); GSSG: Oxidised glutathione (μmol GSSG/mg protein); OSI: Oxidative Stress index (GSH:GSSG); TOS: Total Oxidant Status (µmol H_2_O_2_/mg protein); ROS: Reactive Oxygen Species (µmol DCF/mg protein); TBARS: Thiobarbituric Acid Reactive Substances (μmol MDA/mg protein); GCA: Green Core Area (mm^2^); GIA: Green Inner Area (mm^2^); GOA: Green Outer Area (mm^2^); RCA: Red Core Area (mm^2^); RIA: Red Inner Area (mm^2^); ROA: Red Outer Area (mm^2^); SD: Standard Deviation; CV: Coefficient of Variation (%).

**Table 2:** Correlation coefficients between laser-light backscattering parameters and physicochemical quality traits in Arouquesa beef.

Meat Quality Traits		Green Laser			Red Laser		
		GCA	GIA	GOA	RCA	RIA	ROA
Day 1							
pH_24h_		−0.62 ***	−0.76 ***	−0.71 ***	−0.89 ***	−0.89 ***	−0.91 ***
Day 7							
Colour	L*	0.73 ***	0.60 ***	0.40 *	0.65 ***	0.68 ***	0.52 ***
	a*	0.63 ***	0.56 ***	0.42 **	0.65 ***	0.71 ***	0.64 ***
	b*	0.68 ***	0.63 ***	0.45 **	0.69 ***	0.73 ***	0.68 ***
DL		0.47 **	0.52 **	0.51 **	0.63 ***	0.55 ***	0.65 ***
CL		0.75 ***	0.76 ***	0.61 ***	0.67 ***	0.76 ***	0.71 ***
SL		−0.06	0.15	0.16	0.14	0.17	0.16
SF		0.52 ***	0.41 *	0.37	0.47 **	0.57 ***	0.36 *

pH_24h_: pH 24 h post-mortem; DL: Drip Losses (%); CL: Cooking Losses (%); SL: Sarcomere Length (µm); SF: Shear Force (N/cm^2^); GCA: Green Core Area (mm^2^); GIA: Green Inner Area (mm^2^); GOA: Green Outer Area (mm^2^); RCA: Red Core Area (mm^2^); RIA: Red Inner Area (mm^2^); ROA: Red Outer Area (mm^2^). A positive correlation means that the two variables tend to vary in the same direction, and a negative correlation means that they vary in opposite directions. Statistical significance is indicated as follows: *p* < 0.05 (*), *p* < 0.01 (**), and *p* < 0.001 (***). pH was measured at 24 h and the other traits after 7 days of storage.

**Table 3:** Correlation coefficients between laser-light backscattering parameters and oxidative stress biomarkers in Arouquesa beef.

Trait	Green Laser			Red Laser		
	GCA	GIA	GOA	RCA	RIA	ROA
Day 1						
SOD	0.37 *	0.26	0.19	0.49 **	0.42 **	0.46 **
CAT	−0.08	−0.09	−0.02	−0.07	0.00	0.01
GPx	−0.27	−0.21	−0.17	−0.13	−0.24	−0.02
GST	0.53 ***	0.54 ***	0.31	0.62 ***	0.48 **	0.66 ***
GSH	0.47 **	0.36	0.19	0.45	0.25	0.41
GSSG	0.47 **	0.27	0.34 *	0.60 ***	0.49 **	0.49 **
OSI	−0.27	−0.22	−0.21	−0.38	−0.24	−0.35
TOS	−0.32	−0.51 **	−0.29	−0.47	−0.48 **	−0.31
ROS	0.29	0.07	0.02	0.29	0.13	0.19
TBARS	0.03	−0.09	0.01	−0.06	−0.28	−0.21
Day 7						
SOD	0.13	0.31	0.14	0.25	0.33	0.38 *
CAT	−0.02	0.21	0.04	0.15	0.18	0.28
GPx	−0.23	−0.18	−0.27	−0.18	−0.32	−0.12
GST	−0.13	0.04	−0.12	0.09	0.12	0.30
GSH	−0.08	0.10	−0.11	0.16	0.01	0.28
GSSG	0.41 *	0.36 *	0.44 **	0.44 **	0.42 **	0.36 *
OSI	−0.45 **	−0.31	−0.46 **	−0.33	−0.37 *	−0.19
TOS	−0.47 **	−0.54 ***	−0.28	−0.49 **	−0.51 **	−0.46 **
ROS	0.27	0.35 *	0.26	0.19	0.32	0.37 *
TBARS	0.07	0.24	0.09	0.32	0.24	0.33

SOD: Superoxide Dismutase (U/mg protein); CAT: Catalase (U/mg protein); GPx: Glutathione Peroxidase (nmol NADH/mg protein); GST: Glutathione S-transferase (nmol CDNB/min.mg protein); GSH: Reduced glutathione (μmol GSH/mg protein); GSSG: Oxidised glutathione (μmol GSSG/mg protein); OSI: Oxidative Stress index (GSH:GSSG); TOS: Total Oxidant Status (µmol H_2_O_2_/mg protein); ROS: Reactive Oxygen Species (µmol DCF/mg protein); TBARS: Thiobarbituric Acid Reactive Substances (μmol MDA/mg protein); GCA: Green Core Area (mm^2^); GIA: Green Inner Area (mm^2^); GOA: Green Outer Area (mm^2^); RCA: Red Core Area (mm^2^); RIA: Red Inner Area (mm^2^); ROA: Red Outer Area (mm^2^). A positive correlation means that the two variables tend to vary in the same direction, and a negative correlation means that they vary in opposite directions. Statistical significance is indicated as follows: *p* < 0.05 (*), *p* < 0.01 (**), and *p* < 0.001 (***).

## Data Availability

The raw data supporting the conclusions of this article will be made available by the authors upon request.

## Abbreviations

The following abbreviations are used in this manuscript:

CAT	Catalase
CL	Cooking Losses
CV	Coefficient of Variation
DFD	Dark, Firm, and Dry
DL	Drip Losses
D1	Day one
D7	Day seven
GCA	Green Core Area
GIA	Green Inner Area
GOA	Green Outer Area
GPx	Glutathione Peroxidase
GSH	Reduced glutathione
GSSG	Oxidised glutathione
GST	Glutathione S-transferase
LLBI	Laser-Light Backscattering Imaging
*LTL*	*Longissimus thoracis et lumborum*
OSI	Oxidative Stress Index
pH_24h_	pH 24 h post-mortem
RCA	Red Core Area
RIA	Red Inner Area
ROA	Red Outer Area
ROS	Reactive Oxygen Species
SD	Standard Deviation
SF	Shear Force
SL	Sarcomere Length
SOD	Superoxide dismutase
TBARS	Thiobarbituric Acid Reactive Substances
TOS	Total Oxidant Status
UTAD	University of Trás-os-Montes e Alto Douro

## References

[1] Ponnampalam E.N., Hopkins D.L., Bruce H., Li D., Baldi G., Bekhit A.E. (2017). Causes and Contributing Factors to “Dark Cutting” Meat: Current Trends and Future Directions: A Review. Comp. Rev. Food Sci. Food Safe.

[2] Warner R.D., Wheeler T.L., Ha M., Li X., Bekhit A.E.-D., Morton J., Vaskoska R., Dunshea F.R., Liu R., Purslow P. (2022). Meat Tenderness: Advances in Biology, Biochemistry, Molecular Mechanisms and New Technologies. Meat Sci..

[3] Jacob R. (2020). Implications of the Variation in Bloom Properties of Red Meat: A Review. Meat Sci..

[4] Díaz-Luis A., Díaz F., Diñeiro Y., González-Blanco L., Arias E., Coto-Montes A., Oliván M., Sierra-Sánchez V. (2020). Nuevos indicadores de carnes DFD: Estrés oxidativo, autofagia y apoptosis. ITEA.

[5] Ponnampalam E.N., Kiani A., Santhiravel S., Holman B.W.B., Lauridsen C., Dunshea F.R. (2022). The Importance of Dietary Antioxidants on Oxidative Stress, Meat and Milk Production, and Their Preservative Aspects in Farm Animals: Antioxidant Action, Animal Health, and Product Quality—Invited Review. Animals.

[6] Falowo A.B., Fayemi P.O., Muchenje V. (2014). Natural Antioxidants against Lipid–Protein Oxidative Deterioration in Meat and Meat Products: A Review. Food Res. Int..

[7] Wang J., Wu N., Yao Y., Chen S., Xu L., Zhao Y., Tu Y. (2025). Protein Oxidation and Its Effect on Functional Properties of Livestock Products During the Processing and Storage: A Review. Food Chem. X.

[8] Lawrie R.A., Ledward D.A. (2006). Lawrie’s Meat Science.

[9] Nicolaisen S., Langkabel N., Thoene-Reineke C., Wiegard M. (2023). Animal Welfare During Transport and Slaughter of Cattle: A Systematic Review of Studies in the European Legal Framework. Animals.

[10] Edwards-Callaway L., Flanagan L., Loh H.Y., Sullivan P., Nair M., Engle T. (2025). Characterizing Research Exploring Factors Associated with Dark Cutting, pH, Color, and Glycogen Content in Beef from Grain-Finished or Feedlot-Fed Cattle: A Systematic Mapping Review. Meat Sci..

[11] Crichton S.O.J., Kirchner S.M., Porley V., Retz S., Von Gersdorff G., Hensel O., Sturm B. (2017). High pH Thresholding of Beef with VNIR Hyperspectral Imaging. Meat Sci..

[12] Chen J., Zhang J., Wang N., Xiao B., Sun X., Li J., Zhong K., Yang L., Pang X., Huang F. (2024). Critical Review and Recent Advances of Emerging Real-Time and Non-Destructive Strategies for Meat Spoilage Monitoring. Food Chem..

[13] Nelis J.L.D., Bose U., Broadbent J.A., Hughes J., Sikes A., Anderson A., Caron K., Schmoelzl S., Colgrave M.L. (2022). Biomarkers and Biosensors for the Diagnosis of Noncompliant pH, Dark Cutting Beef Predisposition, and Welfare in Cattle. Comp. Rev. Food Sci. Food Safe.

[14] Hernández-Hernández L., Mahecha-Ledesma L., Angulo-Arizala J., Barragán-Hernández W. (2026). Evaluation of Portable, Low-Cost Techniques for Discriminating the DFD (Dark, Firm, Dry) Condition in Beef. Meat Sci..

[15] Qiao J., Ngadi M.O., Wang N., Gariépy C., Prasher S.O. (2007). Pork Quality and Marbling Level Assessment Using a Hyperspectral Imaging System. J. Food Eng..

[16] Barbin D.F., ElMasry G., Sun D.-W., Allen P. (2012). Predicting Quality and Sensory Attributes of Pork Using Near-Infrared Hyperspectral Imaging. Anal. Chim. Acta.

[17] Fulladosa E., Rubio-Celorio M., Skytte J.L., Muñoz I., Picouet P. (2017). Laser-Light Backscattering Response to Water Content and Proteolysis in Dry-Cured Ham. Food Control.

[18] Peng Y., Zhang J., Wang W., Li Y., Wu J., Huang H., Gao X., Jiang W. (2011). Potential Prediction of the Microbial Spoilage of Beef Using Spatially Resolved Hyperspectral Scattering Profiles. J. Food Eng..

[19] Wu J., Peng Y., Chen J., Wang W., Gao X., Huang H. (2010). Study of spatially resolved hyperspectral scattering images for assessing beef quality characteristics. Guang Pu Xue Yu Guang Pu Fen. Xi.

[20] Pham T.T., Nguyen T.B., Dam M.S., Nguyen L.L.P., Baranyai L. (2024). A Review of the Application of the Laser-Light Backscattering Imaging Technique to Agricultural Products. Agriculture.

[21] Sanchez P.D.C., Hashim N., Shamsudin R., Mohd Nor M.Z. (2020). Potential Application of Laser-Based Imaging Technology in the Quality Evaluation of Agricultural Products: A Review. Adv. Agric. Food Res. J..

[22] Sacarrão-Birrento L., Vitorino A., Pereira A.M., Alves S.P., Borba A., Silva S.R., Carolino N., Venâncio C.A., Almeida A.M.D. (2025). The Portuguese Autochthonous Cattle Breeds and Their Relevance in the Framework of Sustainable Beef Production Systems. Trop. Anim. Health Prod..

[23] Sacarrão-Birrento L., Gomes M.J., Silva S.R., Silva J.A., Moreira D., Vieira R., Ferreira L.M., Pereira P., De Almeida A.M., Almeida J.C. (2022). Growth Performance, Carcass and Meat Traits of Autochthonous Arouquesa Weaners Raised on Traditional and Improved Feeding Systems. Animals.

[24] Silva J.A., Patarata L., Martins C. (1999). Influence of Ultimate pH on Bovine Meat Tenderness During Ageing. Meat Sci..

[25] Carrasco-García A.A., Pardío-Sedas V.T., León-Banda G.G., Ahuja-Aguirre C., Paredes-Ramos P., Hernández-Cruz B.C., Murillo V.V. (2020). Effect of Stress During Slaughter on Carcass Characteristics and Meat Quality in Tropical Beef Cattle. Asian-Australas. J. Anim. Sci..

[26] CIE (1986). Colorimetry.

[27] AMSA (2012). AMSA Meat Color Measurement Guidelines.

[28] Honikel K.O., Tarrant P.V., Eikelenboom G., Monin G. (1987). How to Measure the Water-Holding Capacity of Meat? Recommendation of Standardized Methods. Evaluation and Control of Meat Quality in Pigs.

[29] Cross H.R., West R.L., Dutson T.R. (1981). Comparison of Methods for Measuring Sarcomere Length in Beef Semitendinosus Muscle. Meat Sci..

[30] Durak I., Yurtarslanl Z., Canbolat O., Akyol Ö. (1993). A Methodological Approach to Superoxide Dismutase (SOD) Activity Assay Based on Inhibition of Nitroblue Tetrazolium (NBT) Reduction. Clin. Chim. Acta.

[31] Claiborne A., Greenwald R.A. (1985). Catalase Activity. CRC Handbook of Methods for Oxygen Radical Research.

[32] Massarsky A., Kozal J.S., Di Giulio R.T. (2017). Glutathione and Zebrafish: Old Assays to Address a Current Issue. Chemosphere.

[33] Habig W.H., Jakoby W.B. (1981). [51] Assays for Differentiation of Glutathione S-Transferases. Methods in Enzymology.

[34] Gartaganis S.P., Patsoukis N.E., Nikolopoulos D.K., Georgiou C.D. (2007). Evidence for Oxidative Stress in Lens Epithelial Cells in Pseudoexfoliation Syndrome. Eye.

[35] Erel O. (2005). A New Automated Colorimetric Method for Measuring Total Oxidant Status. Clin. Biochem..

[36] Deng J., Yu L., Liu C., Yu K., Shi X., Yeung L.W.Y., Lam P.K.S., Wu R.S.S., Zhou B. (2009). Hexabromocyclododecane-Induced Developmental Toxicity and Apoptosis in Zebrafish Embryos. Aquat. Toxicol..

[37] Wallin B., Rosengren B., Shertzer H.G., Camejo G. (1993). Lipoprotein Oxidation and Measurement of Thiobarbituric Acid Reacting Substances Formation in a Single Microtiter Plate: Its Use for Evaluation of Antioxidants. Anal. Biochem..

[38] Aldalawi A.A., Suardi N., Ahmed N.M., Al-Farawn M.A.S., Dheyab M.A., Jebur W.I., Kadhim F.J. (2023). Comparison of Wavelength-Dependent Penetration Depth of 532 Nm and 660 Nm Lasers in Different Tissue Types. J. Lasers Med. Sci..

[39] Ruedt C., Gibis M., Weiss J. (2023). Meat Color and Iridescence: Origin, Analysis, and Approaches to Modulation. Comp. Rev. Food Sci. Food Safe.

[40] Bai J., Zhang L., Cai J., Wang Y., Tian X. (2021). Laser Light Backscattering Image to Predict Moisture Content of Mango Slices with Different Ripeness During Drying Process. J. Food Process Eng..

[41] Hughes J.M., Oiseth S.K., Purslow P.P., Warner R.D. (2014). A Structural Approach to Understanding the Interactions Between Colour, Water-Holding Capacity and Tenderness. Meat Sci..

[42] Hughes J., McPhail N., Watkins P., Stark J., Warner R.D. (2023). Increased Light Scattering in Electrically Stimulated Beef Longissimus Muscle Fibres Contributes to the Observed Meat Colour at Grading. Anim. Prod. Sci..

[43] Hui Y.H., Hui Y.H., Nip W.-K., Rogers R. (2001). Meat Science and Applications.

[44] Hughes J.M., Clarke F.M., Purslow P.P., Warner R.D. (2020). Meat Color Is Determined Not Only by Chromatic Heme Pigments but Also by the Physical Structure and Achromatic Light Scattering Properties of the Muscle. Comp. Rev. Food Sci. Food Safe.

[45] Pearce K.L., Rosenvold K., Andersen H.J., Hopkins D.L. (2011). Water Distribution and Mobility in Meat During the Conversion of Muscle to Meat and Ageing and the Impacts on Fresh Meat Quality Attributes—A Review. Meat Sci..

[46] Peng Y., Wu J., Chen J. (2009). Prediction of Beef Quality Attributes Using Hyperspectral Scattering Imaging Technique. Proceedings of the 2009, Reno, NV, USA, 21–24 June 2009.

[47] Ranasinghesagara J., Nath T.M., Wells S.J., Weaver A.D., Gerrard D.E., Yao G. (2010). Imaging Optical Diffuse Reflectance in Beef Muscles for Tenderness Prediction. Meat Sci..

[48] Adebayo S.E., Hashim N., Abdan K., Hanafi M. (2016). Application and Potential of Backscattering Imaging Techniques in Agricultural and Food Processing—A Review. J. Food Eng..

[49] Bekhit A.E.A., Hopkins D.L., Fahri F.T., Ponnampalam E.N. (2013). Oxidative Processes in Muscle Systems and Fresh Meat: Sources, Markers, and Remedies. Comp. Rev. Food Sci. Food Safe.

[50] Dragoev S.G. (2024). Lipid Peroxidation in Muscle Foods: Impact on Quality, Safety and Human Health. Foods.

[51] Ramanathan R., Suman S.P., Faustman C. (2020). Biomolecular Interactions Governing Fresh Meat Color in Post-Mortem Skeletal Muscle: A Review. J. Agric. Food Chem..

[52] Gagaoua M., Warner R.D., Purslow P., Ramanathan R., Mullen A.M., López-Pedrouso M., Franco D., Lorenzo J.M., Tomasevic I., Picard B. (2021). Dark-Cutting Beef: A Brief Review and an Integromics Meta-Analysis at the Proteome Level to Decipher the Underlying Pathways. Meat Sci..

[53] Renerre M., Dumont F., Gatellier P. (1996). Antioxidant Enzyme Activities in Beef in Relation to Oxidation of Lipid and Myoglobin. Meat Sci..

[54] Pouzo L.B., Descalzo A.M., Zaritzky N.E., Rossetti L., Pavan E. (2016). Antioxidant Status, Lipid and Color Stability of Aged Beef from Grazing Steers Supplemented with Corn Grain and Increasing Levels of Flaxseed. Meat Sci..

[55] English A.R., Wills K.M., Harsh B.N., Mafi G.G., VanOverbeke D.L., Ramanathan R. (2016). Effects of Aging on the Fundamental Color Chemistry of Dark-Cutting Beef. J. Anim. Sci..

[56] Hughes J., Clarke F., Li Y., Purslow P., Warner R. (2019). Differences in Light Scattering Between Pale and Dark Beef Longissimus Thoracis Muscles Are Primarily Caused by Differences in the Myofilament Lattice, Myofibril and Muscle Fibre Transverse Spacings. Meat Sci..

[57] McKeith R.O., King D.A., Grayson A.L., Shackelford S.D., Gehring K.B., Savell J.W., Wheeler T.L. (2016). Mitochondrial Abundance and Efficiency Contribute to Lean Color of Dark Cutting Beef. Meat Sci..

[58] Da Fonseca C.A.R., Paltian J., Dos Reis A.S., Bortolatto C.F., Wilhelm E.A., Luchese C. (2019). Na^+^/K^+^-ATPase, Acetylcholinesterase and Glutathione S-Transferase Activities as New Markers of Postmortem Interval in Swiss Mice. Leg. Med..

[59] González-Blanco L., Royo L.J., Diñeiro Y., García-Torres S., Coto-Montes A., Sierra V., Oliván M. (2024). Exploring the miRNAs Profile in Dark-Cutting Beef. Foods.

[60] Rakowska R., Sadowska A., Waszkiewicz-Robak B. (2017). Influence of Pre- and Post-Slaughter Factors on the Reduced Glutathione Content of Beef Muscles. Meat Sci..

[61] Bai X., Tian W., Yin F., Xiao K., Chen Q., Chai R., Ru A., Li J., Zhu C., Zhao G. (2022). Age-Specific Effect on Endogenous Oxidative and Antioxidative Characteristics of Longissimus Thoracis Muscle of Yak During Early Postmortem Period. Food Chem..

[62] Mitacek R.M., Ke Y., Prenni J.E., Jadeja R., VanOverbeke D.L., Mafi G.G., Ramanathan R. (2019). Mitochondrial Degeneration, Depletion of NADH, and Oxidative Stress Decrease Color Stability of Wet-Aged Beef Longissimus Steaks. J. Food Sci..

[63] Faustman C., Cassens R.G., Schaefer D.M., Buege D.R., Williams S.N., Scheller K.K. (1989). Improvement of Pigment and Lipid Stability in Holstein Steer Beef by Dietary Supplementation with Vitamin E. J. Food Sci..

[64] Castaño Ledesma M.S., Palma G.A., Borsarelli C.D., Coria M.S. (2025). Comparative Analysis of Feeding Strategies and Post Mortem Aging Time on the Oxidative Status and Color of the Longissimus Thoracis et Lumborum Muscle in Braford Steers. Front. Anim. Sci..

[65] Domínguez R., Pateiro M., Gagaoua M., Barba F.J., Zhang W., Lorenzo J.M. (2019). A Comprehensive Review on Lipid Oxidation in Meat and Meat Products. Antioxidants.

[66] Cavalcante C.L., Gonza M.M.C., Lopes B.G., Savian T.V., Sartori A.G.D.O., Pinto J.S.D.S., Contreras Castillo C.J. (2025). Impact of Ultimate pH on Oxidative Stability and Warmed-Over Flavor in Aged Beef After Grilling and Reheating. Int. J. Gastron. Food Sci..

